# Inferring muscular ground patterns in Bivalvia: Myogenesis in the scallop *Nodipecten nodosus*

**DOI:** 10.1186/s12983-015-0125-x

**Published:** 2015-12-02

**Authors:** Jorge A. Audino, José Eduardo A. R. Marian, Alen Kristof, Andreas Wanninger

**Affiliations:** Department of Zoology, University of São Paulo, Rua do Matão, Travessa 14, 101, 05508-090 São Paulo, Brazil; Department of Integrative Zoology, University of Vienna, Althanstrasse 14, 1090 Vienna, Austria

**Keywords:** Bivalve, EvoDevo, Evolution, Morphology, Musculature, Morphogenesis, Ontogeny, Pectinid

## Abstract

**Background:**

Myogenesis is currently investigated in a number of invertebrate taxa using combined techniques, including fluorescence labeling, confocal microscopy, and 3D imaging, in order to understand anatomical and functional issues and to contribute to evolutionary questions. Although developmental studies on the gross morphology of bivalves have been extensively pursued, organogenesis including muscle development has been scarcely investigated so far.

**Results:**

The present study describes in detail myogenesis in the scallop *Nodipecten nodosus* (Linnaeus, 1758) during larval and postmetamorphic stages by means of light, electron, and confocal microscopy. The veliger muscle system consists of an anterior adductor muscle, as well as four branched pairs of striated velum retractors and two pairs of striated ventral larval retractors. The pediveliger stage exhibits a considerably elaborated musculature comprising the velum retractors, the future adult foot retractor, mantle (pallial) muscles, and the anterior and posterior adductors, both composed of smooth and striated portions. During metamorphosis, all larval retractors together with the anterior adductor degenerate, resulting in the adult monomyarian condition, whereby the posterior adductor retains both myofiber types. Three muscle groups, i.e., the posterior adductor, foot retractor, and pallial muscles, have their origin prior to metamorphosis and are subsequently remodeled.

**Conclusions:**

Our data suggest a dimyarian condition (i.e., the presence of an anterior and a posterior adductor in the adult) as the basal condition for pectinids. Comparative analysis of myogenesis across Bivalvia strongly argues for ontogenetic and evolutionary independence of larval retractors from the adult musculature, as well as a complex set of larval retractor muscles in the last common bivalve ancestor.

**Electronic supplementary material:**

The online version of this article (doi:10.1186/s12983-015-0125-x) contains supplementary material, which is available to authorized users.

## Background

Ontogeny of bivalve gross anatomy has been widely investigated during the past two centuries and bivalve larvae have been used as models in studies dealing with diverse biological questions (e.g., [1, 2]). Developmental studies in this class-level taxon of mollusks have been performed for numerous species using light and electron microscopy, and a large bulk of data on larval general morphology is available (e.g., [3, 4]). Nevertheless, several gaps of knowledge on bivalve development remain, especially on myogenesis, neurogenesis, and morphogenesis of other organ systems [5–7].

Morphological investigations have significantly contributed to understanding the function of animal organ systems and the evolution of phenotypic diversity. In particular, the relevance of morphology for EvoDevo approaches has been highlighted as being indispensable for reconstruction of phenotypic ground patterns and character evolution (see [8]). Modern methods including fluorescence staining combined with confocal microscopy and three-dimensional reconstruction have been successfully applied in the last decade to study invertebrate organogenesis, including muscular development (see [9, 10] for reviews). Such techniques, particularly phalloidin staining, represent a powerful tool for studies into the microanatomy and development of the often complex musculature, in particular of minute organisms including invertebrate larvae.

Within Mollusca, a solid database exists for a number of class-level taxa including the Gastropoda (e.g., [11–13]), Scaphopoda [14], Polyplacophora [15], and Neomeniomorpha (= Solenogastres) [16, 17]. The data generated have yielded important insights into developmental and evolutionary pathways within the phylum such as, for example, the emergence of the neomeniomorph worm-like body from a much more complex ancestor (see [7] for extensive review on the current state-of-the-art of molluscan EvoDevo). Despite this recent progress, data on myogenesis are particularly scarce for the second-largest molluscan class, the Bivalvia. Thereby, the most complete data are currently available for two species only, namely the mytilid *Mytilus trossulus* [18, 19] and the teredinid shipworm *Lyrodus pedicellatus* [20].

Pectinidae (scallops) comprises a very diverse family of pteriomorphian epifaunal bivalves, but despite a broad knowledge on larval anatomy and preliminary characterization of larval muscles in *Pecten maximus* [21], no further details on scallop muscle development are available. To inject novel data into the discussion of shared and diverging morphological characters within Pectinidae and across Bivalvia, and to reconstruct potential bivalve larval ground patterns, the present study provides a detailed description of myogenesis in the scallop *Nodipecten nodosus* (Linnaeus, 1758) during larval and postmetamorphic development.

## Results

### Nomenclature

Herein, designation of larval orientation and body axes are used in accordance with comparative lophotrochozoan larval anatomy (e.g., [22]). In larvae, anterior corresponds to the position of the apical tuft, posterior to the opposite region, and ventral is defined by the position of the foot (see also [20]), i.e., for veliger larvae we consider herein the velum region as anterior and the hinge as posterior. We follow this nomenclature for all larval muscles but not for the future adult “anterior adductor” (larval dorsal) and “posterior adductor” (larval ventral), respectively, to avoid confusion when comparing larval and adult conditions. In postmetamorphic bivalves, body rotation may result in deep changes in morphology and, consequently, in nomenclatorial conflicts. Consensually, adult axes designation in bivalves is commonly such that the mouth position defines anterior and the shell hinge dorsal (with the opposite as ventral).

### Myogenesis

**Fig. 1:**
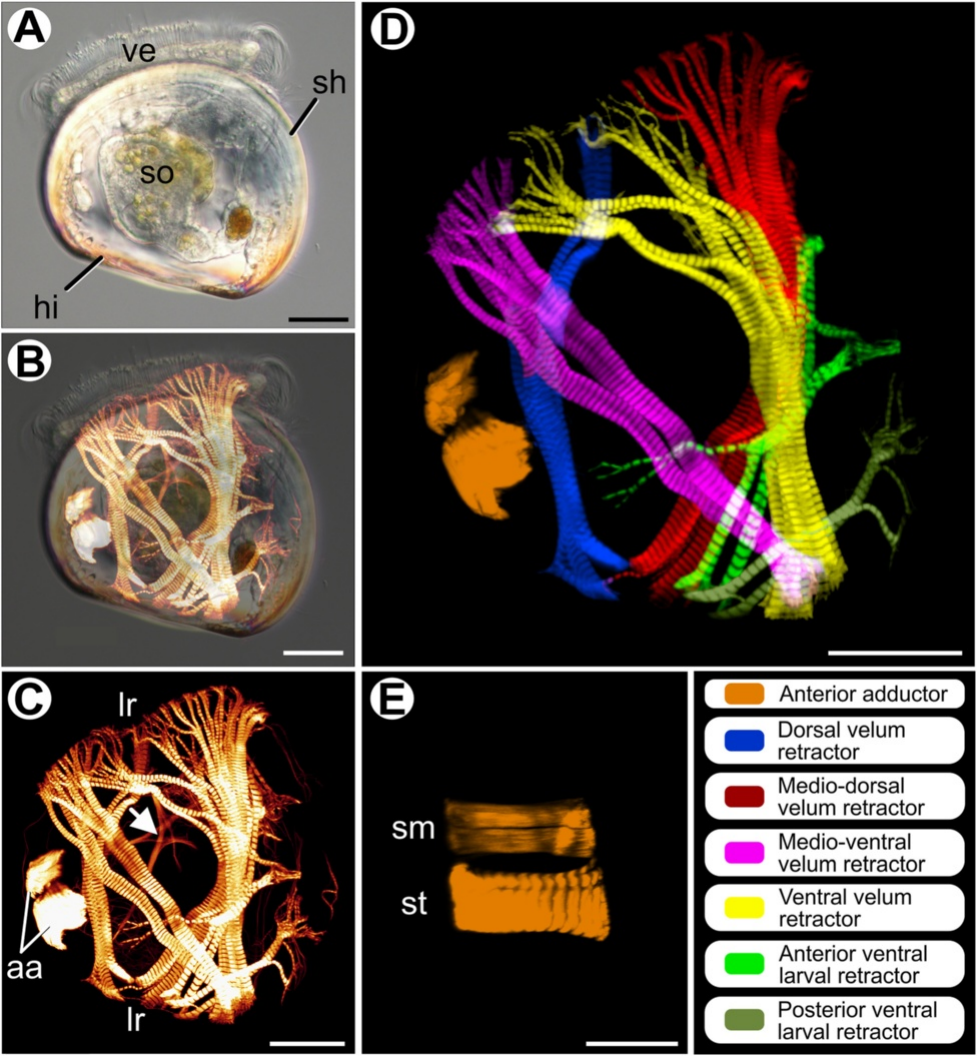
Myoanatomy of *Nodipecten nodosus* veliger larvae. Anterior to the top in all images, ventral to the right in (**a**-**d**). Scale bars: 20 μm. **a** Specimen observed by light microscopy using differential interference contrast. Lateral view. **b** Same image from (**a**) combined with confocal image produced by phalloidin staining to visualize the relative position of larval muscle systems. **c** Confocal micrograph of larval musculature; arrow points to smooth muscles in the median region of the digestive tract. Lateral view. **d** 3D reconstruction of veliger musculature showing anterior adductor, ventral larval retractors, and velum retractors. Lateral view. **e** Detail of a 3D reconstruction of the anterior adductor of a veliger larva with their smooth and striated units. Dorsal view. Abbreviations: *aa*, anterior adductor; *hi*, hinge; *lr*, larval retractors; *sh*, shell; *sm*, smooth unit; *so,* stomach; *st*, striated unit; *ve*, velum

Veliger larvae of *Nodipecten nodosus* (Fig. 1a) exhibit a massive and highly complex musculature comprised of distinct muscle groups (Fig. 1b, c, and Additional file 1). In the median region of the digestive tract, thin muscle bundles comprised of apparently smooth fibers are present and are possibly associated with the stomach (Fig. 1c). The larval velum retractors, responsible for the retraction of the velum into the pallial cavity, form the most prominent muscle group and are organized in four branched pairs containing exclusively striated fibers (Fig. 1c, d). Symmetrically distributed on both sides of the body, these velum retractors are attached to the shell in the region of the hinge and, except for the dorsal velum retractors, they become profoundly branched where the fibers reach the velum (Fig. 1d). The dorsal velum retractors are dorsally attached to the shell, near the anterior adductor, with few branches running anteriorly to the median portion of the velum (Fig. 1d). The medio-dorsal velum retractors are medially attached, profusely branching in the ventral region of the velum (Fig. 1d). The medio-ventral retractors cross the body obliquely along the dorso-ventral axis, branching in the dorsal portion of the velum (Fig. 1d). Finally, the ventral velum retractors are ventrally attached and become divided into two main groups of branches, each one reaching the ventral and dorsal portions of the larval velum, respectively (Fig. 1d).

Two additional, distinct pairs of muscles, termed herein ventral larval retractors, also formed by striated fibers, are found attached in a more ventral region of the larva (Fig. 1d). The posterior ventral larval retractors branch before reaching the ventral body wall, while the anterior ventral larval retractor has an additional branch running towards the median portion of the larval body (Fig. 1d). The anterior adductor muscle is already present at this stage and comprises two portions: a slightly thinner one, composed of smooth fibers, that is situated more anteriorly, and a larger one composed of striated fibers that is more posteriorly located (Fig. 1e).

**Fig. 2:**
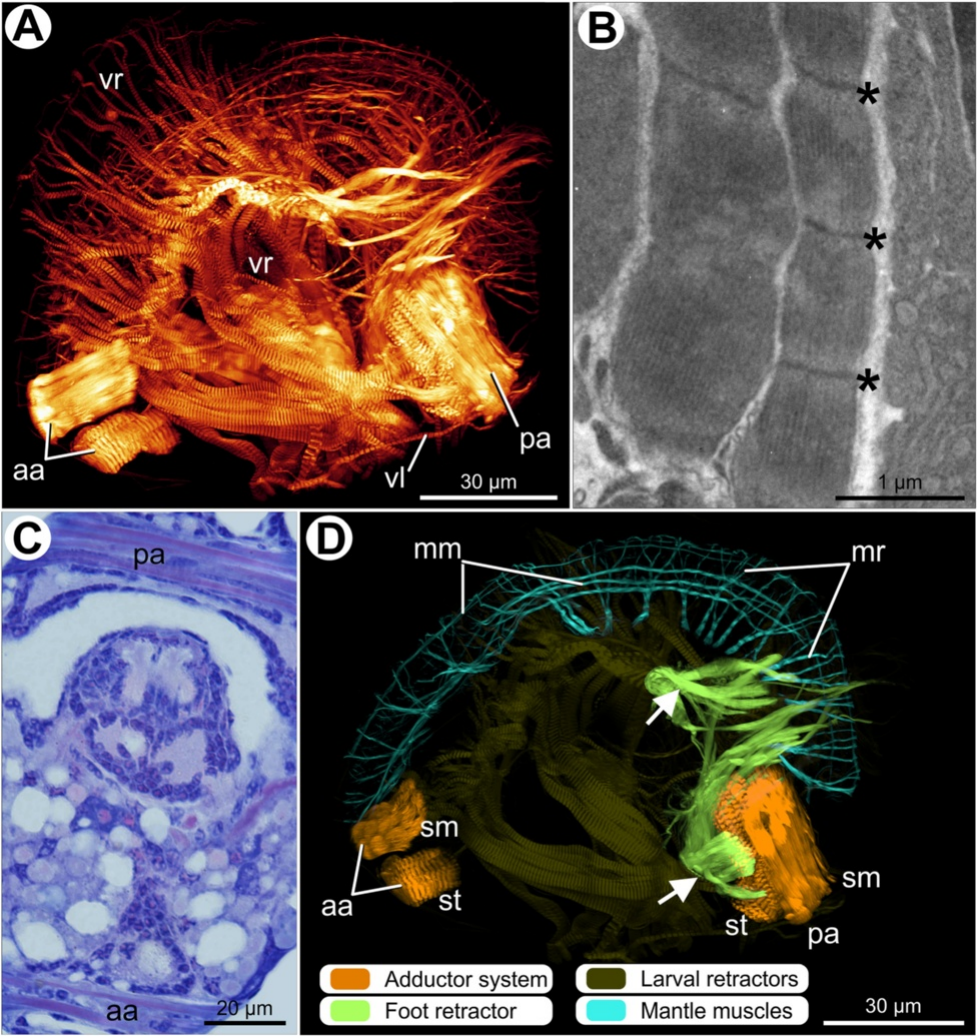
Myoanatomy of pediveligers of *Nodipecten nodosus*. Anterior to the top and ventral to the right in (**a**), (**b**), and (**d**). Ventral to the top in (**c**). **a** Confocal micrograph of the musculature of a pediveliger larva. Lateral view. **b** Transmission electron micrograph of the striated fibers of the velum retractor; asterisks indicate the Z-lines defining sarcomere limits. **c** Light micrograph of a semithin cross-section through a pediveliger showing the posterior and anterior adductor fibers. Toluidine blue and basic fuchsin (TB). **d** 3D reconstruction of the musculature of a pediveliger larva showing mantle muscles, adductors formed by striated and smooth portions, and foot retractor with combined arrangement of bundles originating from the median region (arrowhead) and from the posterior adductor region (arrow). Lateral view. Larval retractors (i.e., velum retractors and ventral retractors) are depicted in dark green to allow for prominent visualization of other muscle groups. Abbreviations: *aa*, anterior adductor; *mm*, mantle margin-parallel muscles; *mr*, mantle retractors; *pa*, posterior adductor; *sm*, smooth portion; s*t*, striated portion; *vl*, ventral larval retractor; *vr*, velum retractors

The larval muscle system of *Nodipecten nodosus* undergoes a dramatic increase in number and size of fibers during development into the pediveliger stage (Fig. 2a; Additional file 2). The velum retractors become intensely branched (Fig. 2a) and their striated muscles occupy a major portion of the visceral mass, where the original pairs can no longer be recognized individually. The cross-striated pattern of these retractors is very prominent, containing striated fibers organized in sarcomeres with evident Z-lines (sarcomere’s limits) (Fig. 2b). In contrast to the notable increase in larval musculature, the ventral larval retractors neither exhibit additional bundles nor did we observe an increase in size compared to the velum retractors (Fig. 2a). The anterior adductor is still present and its morphology is the same as that described for the veligers (Fig. 2d). A posterior adductor is already developed at this stage and, similar to the anterior adductor, also consists of striated and smooth portions (Fig. 2c, d). However, in this case, both portions are closely grouped into one single column (Fig. 2c, d). Anterior and posterior adductors interconnect the shell valves, crossing the body laterally (Fig. 2c). The developing foot retractor of the pediveligers seems to contain smooth muscles with two different attachment sites: ventral bundles from the medio-ventral region and ventral bundles from the posterior adductor region, respectively (Fig. 2d). Both muscle groups extend into the foot. This arrangement gives rise to the *anlage* of the foot musculature.

The mantle margin of the pediveliger exhibits muscles running along its extension (Fig. 2d), a condition not present in earlier stages. Such muscles are comprised of margin-parallel fibers (apparently including striated and smooth fibers) and retractor bundles (striated fibers only), indicating the onset of development of the mantle retractors (Fig. 2d).

**Fig. 3:**
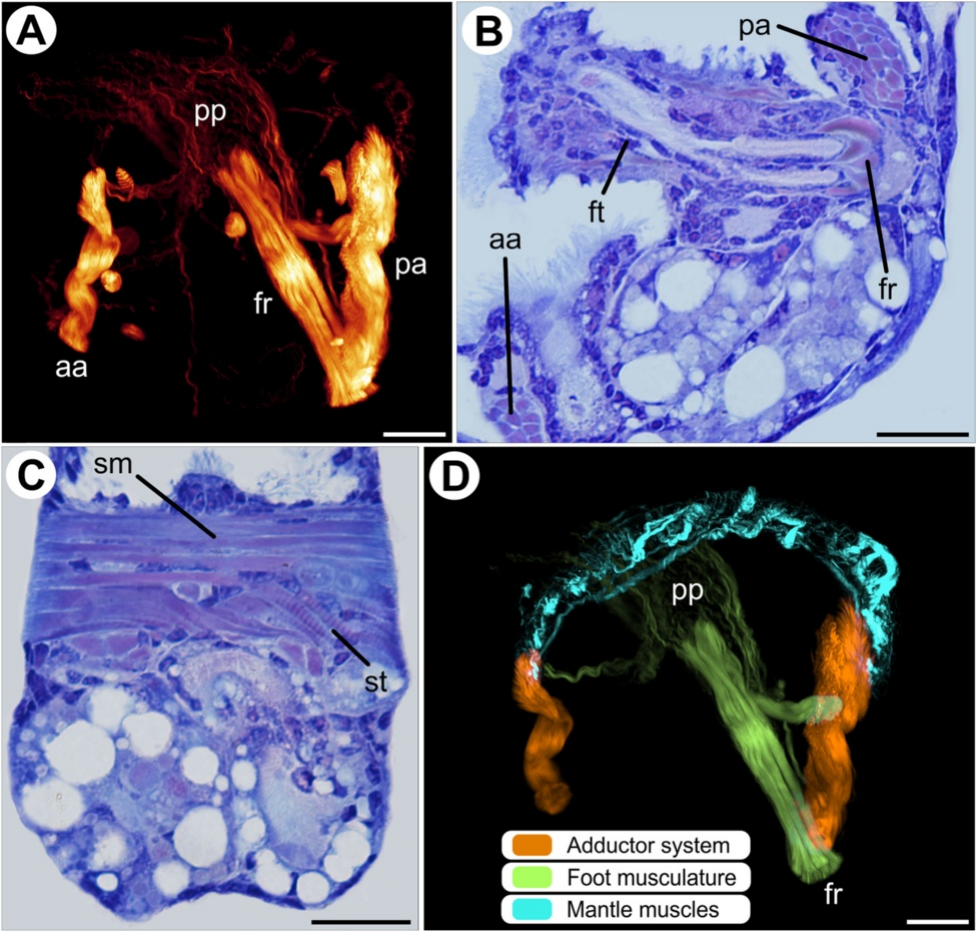
Myoanatomy of *Nodipecten nodosus* postlarvae shortly after metamorphosis. Ventral is to the top in all images (postmetamorphic orientation, i.e., larval anterior), anterior is to the left in (**a**) and (**d**). Scale bars: 20 μm. **a** Confocal micrograph revealing that the larval retractors (velum retractors and ventral larval retractors) have been completely resorbed; the anterior adductor has started to degenerate. Lateral view. **b** Light micrograph of a longitudinal section through a postlarval specimen showing the remaining anterior adductor, the posterior adductor, and the foot retractor. Toluidine blue and basic fuchsin (TB). **c** Cross-section of a postlarval specimen showing the posterior adductor muscle comprised of smooth and striated portions. TB. **d** 3D reconstruction of the postmetamorphic musculature showing mantle muscles, adductors formed by striated and smooth portions, and foot musculature with foot retractor and pedal plexus. Lateral view. Abbreviations: *aa*, anterior adductor; *fr*, foot retractor; *ft*, foot; *pa*, posterior adductor; *pp*, pedal plexus; *sm*, smooth portion; *st*, striated portion

After settlement, metamorphosis leads to rather abrupt modifications of the myoanatomy of *Nodipecten nodosus*. In early metamorphic stages, the larval retractors (velum retractors and ventral larval retractors) and the anterior adductor are under degeneration (Fig. 3a, b). Juveniles show a large posterior adductor muscle with both smooth and striated fibers (Fig. 3c). The foot musculature, composed of a foot retractor and a pedal plexus, is more prominent and exclusively composed of smooth fibers (Fig. 3b, d). The pedal plexus is formed by several long, thin bundles in the distal region of the foot (Fig. 3d). The foot retractor is a compact muscle mass mainly formed by fibers attached to the shell in the posterior adductor region (Fig. 3b, d), i.e., in the same region where one of the ventral bundles of the foot retractor is present in the pediveliger stage (arrow in Fig. 2d). The other muscle bundle composing the foot retractor in pediveligers (from the medio-ventral region; arrowhead in Fig. 2d) is lost. After metamorphosis, the foot is shifted towards the adult anterior region, thus the foot retractor crosses the adult anterior-posterior body axis. Muscles at the postmetamorphic mantle margin are similar to those present in the pediveliger stage, including margin-parallel and retractor bundles (Fig. 3d, Additional file 3).

**Fig. 4:**
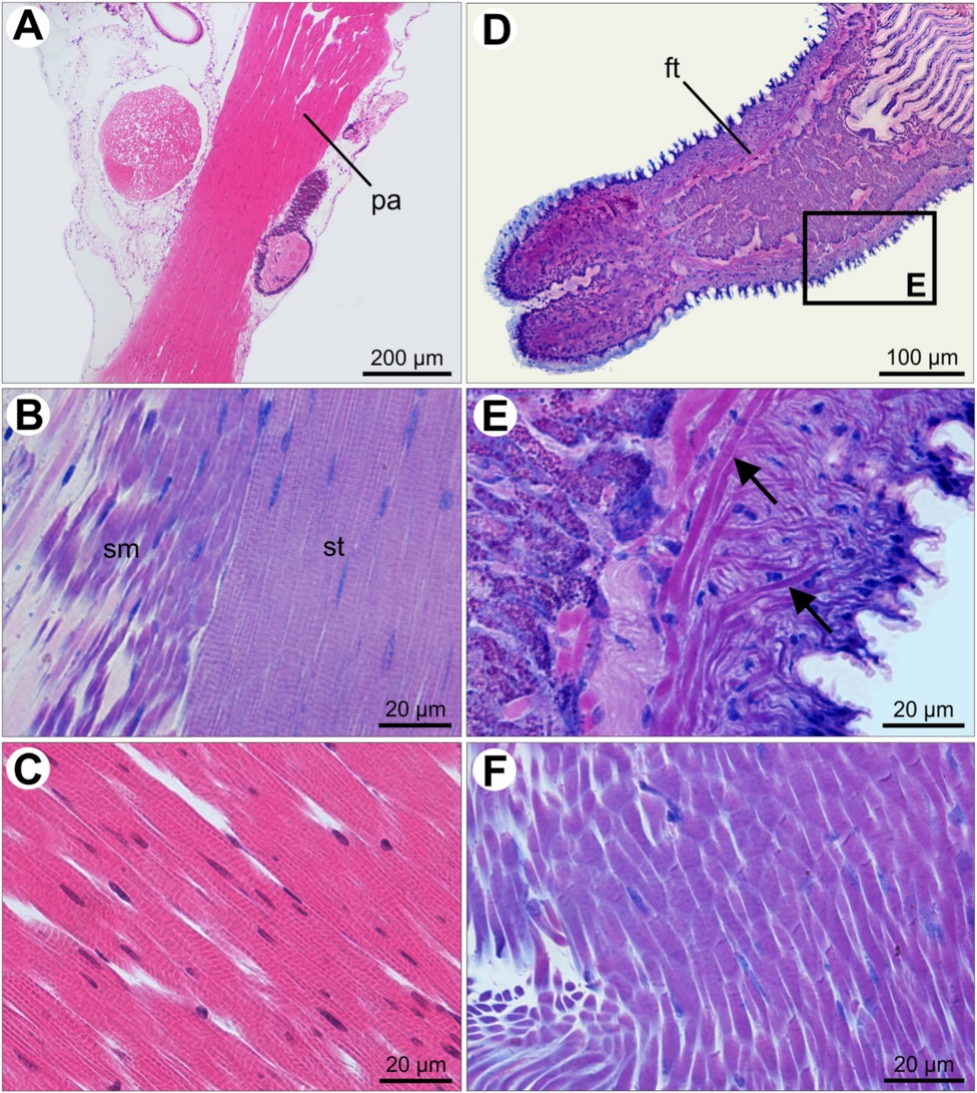
Histological sections of muscular subsets of *Nodipecten nodosus* juveniles. **a** Posterior adductor in longitudinal section. Hematoxylin and eosin (HE). **b** Detail of adjacent smooth and striated portions of the posterior adductor. Toluidine blue and basic fuchsin (TB). **c** Detail of striated bundles of the posterior adductor. HE. **d** Longitudinal section of the foot. TB. **e** Detail of the inset shown in (**d**) with smooth fibers (arrows) spreading over the foot and forming the pedal plexus. TB. **f** Smooth muscles of the foot retractor. TB. Abbreviations: *ft*, foot; *pa*, posterior adductor; *sm*, smooth portion; *st*, striated portion

Several weeks after metamorphosis, juvenile *Nodipecten nodosus* acquire the adult shell features (e.g., color, shape, and sculpture). At this stage, juveniles are 2–5 mm in length and the internal organs are in rapid differentiation and growth. The monomyarian condition is established, since the anterior adductor has completely degenerated. From now on, the enlarged posterior adductor forms the predominant muscle of the adult scallop muscular bodyplan (Fig. 4a). The combined arrangement of smooth and striated fibers in the posterior adductor persists (Fig. 4b), although its major portion becomes striated (Fig. 4c). Within the foot, several smooth muscle bundles form the pedal plexus, allowing for a diverse array of foot movements (Fig. 4d, e). The foot retractor is now formed by dense bundles of smooth muscles (Fig. 4f), similar to the condition described for the early juveniles shortly after metamorphosis.

## Discussion

### General notes on the development of pectinid bivalves

Embryonic and larval development in pectinids has been studied for commercial purposes, yielding a bulk of data on shell development and gross morphology (e.g., [23–27]). However, details on organogenesis, including neuromuscular development, are rare and are only available for *Pecten maximus* and *Argopecten purpuratus*, which have been studied using light and electron microscopy techniques [21, 28]. Myogenesis in *Nodipecten nodosus*, as described herein, represents a significant input of anatomical features and the dynamics of their ontogenetic establishment and remodeling during scallop development and thus contributes significantly to a broader understanding of myogenesis and larval myoanatomy in bivalves.

### Velum retractor muscles

A striated pattern of the velum retractors is present in veligers of *Mytilus edulis* [29], *Crassostrea virginica* [30], *Mytilus trossulus* [18, 19], and *Crassostrea gigas* [6]. A different condition is found in *Lyrodus pedicellatus* [20], where velum retractors are supposedly formed by smooth fibers, although detailed electron microscopy data are lacking. Similarly, smooth larval retractors are present in *Lasaea adansonii* [31], although this species does not exhibit a true velum during ontogeny. The striated velum retractors in veligers of *Nodipecten nodosus* are similar to those described for the scallop *Pecten maximus* [21] (see Table 1 for nomenclatorial correspondence and suggested homologies of individual retractor muscles). The branching pattern is similar in both species, including the major division of the ventral velum retractor into two distinct branches. In addition, positions of insertion in the velum for each retractor of *N. nodosus* are in accordance with descriptions provided for *P. maximus*. Nevertheless, in the latter species, the medio-ventral velum retractor is posteriorly bifurcated near the shell, a condition not observed in *N. nodosus*.

**Table 1:** Bivalve larval retractors and the terms used in the literature and herein. Muscles in the same line indicate proposed homology across species. Data based on the study herein (*Nodipecten nodosus*) as well as on Cragg [21] (*Pecten maximus*) and Wurzinger-Mayer et al. [20] (*Lyrodus pedicellatus*)

	*Nodipecten nodosus*	*Pecten maximus*	*Lyrodus pedicellatus*
Velum retractors	Dorsal velum retractor	Velum retractor 1	Dorsal velum retractor
	Medio-dorsal velum retractor	Velum retractor 2	Ventral velum retractor
	Medio-ventral velum retractor	Velum retractor 3	-
	Ventral velum retractor	Velum retractor 4	-
Ventral larval retractors	Posterior ventral larval retractor	Posterior retractor 1	Ventral larval retractor
	Anterior ventral larval retractor	Posterior retractor 2	-
	-	Posterior retractor 3	-

While four pairs of velum retractors were observed in *Nodipecten nodosus, Pecten maximus* [21], *Argopecten purpuratus* [28], and *Crassostrea gigas* [6], three pairs are present in *Mytiuls trossulus* [19] and *Lasaea adansonii* [31], and only two pairs in *Lyrodus pedicellatus* (not counting the unpaired accessory velum retractor of this species; [20]). Comparisons to velum retractors of *M. trossulus* and *L. adansonii* are difficult because muscle position and insertion sites are not clear. According to the thorough description of the velum retractors in *L. pedicellatus* larvae, the dorsal and ventral velum retractor of this species may correspond to the dorsal and medio-dorsal velum retractors of *N. nodosus*, respectively (Table 1). In both species, the dorsal velum retractors are dorsally attached, pass very close to the anterior adductor, and eventually reach the dorso-median region of the velum. The ventral velum retractors of *L. pedicellatus* and the medio-dorsal velum retractors of *N. nodosus* are attached to the shell slightly ventral to the dorsal velum retractor. These muscles branch and reach the velum in its most ventral portion.

### Ventral larval retractors

Two pairs of shell-anchored ventral larval retractors are present in *Nodipecten nodosus* and in veligers of *Pecten maximus* (“posterior retractors”, [21]). Notwithstanding, a third pair of larval retractors, occupying a more anterior region, has been described for *P. maximus* [21]. Similarly, three pairs of ventral larval retractors are present in the mussel *Mytilus trossulus* (there termed “larval protractors”; [19]). In contrast, a single pair of ventral larval retractors was described for the heterodonts *Lyrodus pedicallatus* [20] and *Dreissena polmorpha* [32], possibly corresponding to the posterior ventral larval retractor of *N. nodosus* (Table 1). In all bivalve species studied so far, this particular muscle group degenerates before metamorphosis, thus representing an ephemeral (transitory) muscle group in bivalve myogenesis [19–21].

### Adductor system

As in *Nodipecten nodosus*, the development of the anterior adductor and velum retractors also occurs in early veliger stages of *Argopecten purpuratus*, *Argopecten irradians*, and *Pecten maximus* [2, 28, 33]. Apparently, the development of the anterior adductor prior to the posterior adductor is a common feature among bivalves (e.g., [19, 20, 30]). The dimyarian condition in early *N. nodosus* development confirms the ancestral condition of two adductors for pectinids and possibly the entire Bivalvia. The anterior adductor of *N. nodosus* comprises two portions, one composed of smooth fibers and the other of striated fibers; a similar morphology was found in *A. purpuratus* and *P. maximus* [21, 28].

### Foot musculature

Previously, it had been suggested that the foot of pectinid pediveliger larvae might originate from ventral larval retractors (“posterior retractors”, [2]). However, such a condition was not found during *Nodipecten nodosus* myogenesis, where both muscle groups are present at the pediveliger stage (Fig. 2a, d). Larvae of *Lyrodus pedicellatus* exhibit the developing foot retractor in a ventral position, close to the posterior adductor *anlage*, possibly originating at the ventral branches of some velum retractors [20]. Our results indicate that the foot retractor in *N. nodosus* is formed by combined muscles originating from two distinct positions. One bundle is ventrally attached to the shell at the posterior adductor region, close to the hinge line where some velum retractors are attached. These bundles might correspond to the foot retractor *anlagen* observed in *L. pedicellatus* and *Lasaea adansonii*, where the foot retractor also originates in a similar position [31]. The second group forming the foot retractor in *N. nodosus* seems to emerge from an anterio-ventral position, close to the median portion of the body. Our results strongly contradict the previous assumption that the (adult) scallop foot retractor derives from larval retractors [2], also because the muscle bundles forming the foot retractor in pediveligers of *N. nodosus* are composed of smooth fibers, while the larval retractors are striated. In addition, all striated larval retractors are lost during metamorphosis and are thus not incorporated into the adult bodyplan, resulting in entirely independent and *de novo* formation of the adult foot musculature.

### Mantle musculature

Muscles associated with the pallial margin have until now received little attention in studies on bivalve development. At the pediveliger stage, smooth fibers are supposed to be found near the mantle folds in *Pecten maximus* [21], and longitudinal smooth muscles are present at the mantle rim of *Lasaea adansonii*, together with several mantle retractors [31]. The larval mantle musculature in *Lyrodus pedicellatus* includes U-shaped muscles, which might correspond to the margin-parallel muscles observed in *Nodipecten nodosus*, as well as finger-shaped muscles, with no obvious correspondence to other bivalve muscles identified so far [20]. The branched retractors and margin-parallel bundles that spread along the mantle margin of *N. nodosus* pediveligers are retained after metamorphosis and they suggest potential retraction of the entire margin and slight alteration of its form by muscular contractions [34].

### Postmetamorphic muscle development

Bivalve metamorphosis has been extensively studied in several species on gross morphological level, some of them including descriptions of organ systems in the larva (e.g., [32, 35–42]). Changes in muscle system organization during metamorphosis are well known only for some species, i.e., *Mytilus edulis* [29], *Mytilus trossulus* [19], and *Ostrea edulis* [43]. In *Nodipecten nodosus*, metamorphosis initiates a dramatic simplification of the musculature: the major portion of striated larval muscles, including all velum retractors and ventral larval retractors, is resorbed. In addition, the anterior adductor starts to degenerate and only the mantle and the posterior adductor exhibit striated muscles in postlarval stages. The postmetamorphic foot musculature is organized into a foot retractor and a pedal plexus, both composed exclusively of smooth fibers. The muscle bundle in the ventral region, close to the median portion of the velum retractors, is lost; the adult foot retractor is formed by bundles that have originated in the posterior ventral region during the pediveliger stage.

The presence of both striated and smooth muscles in the posterior adductor, as observed in *Nodipecten nodosus*, is a typical feature of the scallop musculature [44, 45]. The striated subset increases relative to the smooth one during postmetamorphic development. Oysters also have a posterior adductor formed by two portions; however, in these bivalves the fibers are obliquely striated, in contrast to the cross-striated pattern found in scallops [46, 47]. Interestingly, after metamorphosis, striated muscles appear to be absent in bivalves outside the Pectinidae [19].

### Reconstructing ancestral muscular bodyplan features in Bivalvia

Details on bivalve myogenesis are only slowly emerging, but the data currently available already allow for comparisons and initial attempts to cast some light on the ground patterns of bivalve muscle morphogenesis and the evolution of larval muscular features. Recent phylogenetic hypotheses provide a suitable framework to interpret these issues within an evolutionary context (e.g., [48]).

A muscle ring underneath the developing velum is present in *Lyrodus pedicellatus* and *Mytilus trossulus* (but apparently absent in *Nodipecten nodosus*) as well as in most other molluscan larvae (see [22]), suggesting that it was also part of the bivalve larval ground pattern (see below and [10, 19, 20]). The larval retractor system is the most conspicuous muscular system in the bivalve larva and it addresses two main issues concerning the ancestral reconstruction of larval musculature: (i) The distribution and developmental dynamics of striated and non-striated fibers during myogenesis. While pteriomorphian bivalves such as *N. nodosus*, *M. trossulus*, and *Crassostrea gigas* have striated velum retractors ([6, 19]; present study), heterodont species, for example, *Lasaea adansonii* and *L. pedicellatus*, apparently only exhibit smooth velum retractors [20, 31]. (ii) The number of velum retractor pairs that varies between bivalve species [20] and the number of ventral larval retractors that may range from one to three (see [20]), including two observed in *N. nodosus*. This varying number of larval retractors in the few species investigated, together with the lack of information on the majority of bivalve subclades (including the putatively basal protobranchs), renders any assessment concerning the ancestral number and type of larval retractors highly speculative. In any case, our results and previous data strongly suggest that larval retractors profusely branch during larval development and undergo complete degeneration during metamorphosis, demonstrating the ontogenetic, and most likely also evolutionary, independence of the larval retractor systems from the adult bivalve musculature.

The development of the bivalve adductor systems reveals surprising differences in myofiber constitution during larval and adult stages, also in number of adductors and the dynamics of their formation and degeneration. A dimyarian condition is prevalent in bivalve myogenesis, at least in all species studied so far [20], even in indirect developers that exhibit a so-called pericalymma larva instead of a trochophore such as the protobranchs *Nucula delphinodonta* and *Yoldia limatula* [49, 50]. Thereby, the anterior adductor is always formed prior to the posterior one, and both adductors consist of two distinct subunits (see [20, 51]). Degeneration of the anterior adductor during metamorphosis appears typical for many pteriomorphian bivalves, confirming the notion that these bivalves also stem from an ancestor that had two adductor systems as adult. Overall, however, the assessment of homology hypotheses for adductor systems across the Bivalvia still represents a complicated issue, since different types of myofibers are present in different taxa and the adductors may be formed *de novo* during development, as observed in the freshwater mussel *Anodonta cellensis*, where the (single) adductor of the glochidium larva degenerates and the adult adductor system appears to develop independently [52].

## Conclusions

A comparison of myogenesis across Mollusca reveals important insights into shared and diverging features of the larval muscular bodyplan of the various class-level taxa. As such, a prototroch muscle ring is present in bivalves [19, 31], gastropods [11, 13], polyplacophorans [15], and aplacophorans [16, 53], and thus appears to be a feature of the larval muscular architecture of the last common molluscan ancestor [10]. By contrast, explicit larval retractors with distinct shell insertion sites have so far only been found in bivalves and gastropods but are absent in scaphopods, polyplacophorans, and, obviously, the shell-less aplacophorans [14, 16, 54], and thus might have evolved either independently in the respective conchiferan clades or at the base of Conchifera with secondary loss in the scaphopods.

The recent findings of a highly complex, polyplacophoran-like myoanatomy in larvae of a neomeniomorph aplacophoran [16, 17] and the description of a fossil aplacophoran with seven shell plates [55] argue for a complex last common aculiferan ancestor, most likely with seven-fold seriality in its dorsoventral musculature and, possibly, seven shell plates. Given the recently suggested basal dichotomy in deep molluscan phylogeny [56, 57] that proposes a split of the phylum into Aculifera (Polyplacophora, Neomeniomorpha, Chaetodermomorpha) and Conchifera (all remaining taxa), it remains unclear whether the ur-aculiferan condition of a complex body musculature was also present in the ur-mollusk. In any case, however, the comparative data presently available dramatically highlight the strong dynamics that act during the ontogenetic establishment of the musculature in the various molluscan representatives and reflect the evolutionary plasticity in the myogenetic and myoanatomical pathways at all phyletic levels within this most diverse lophotrochozoan phylum.

## Methods

### Animals

Specimens of *Nodipecten nodosus* at different developmental stages, i.e., veliger, pediveliger, postmetamorphic, and juvenile, were obtained from the scallop farm *Institute of Eco-Development from Baía de Ilha Grande* (IED-BIG), Rio de Janeiro, Brazil. Samples were removed from artificial hatcheries and anesthetized with 7.5 % MgCl_2_ prior to fixation.

### Light (LM) and Transmission Electron Microscopy (TEM)

Specimens were fixed for 3 h at 4 °C in a modified Karnovsky solution (2 % paraformaldehyde + 2,5 % glutaraldehyde in 0.1 M sodium cacodylate buffer at pH 7.4 and 1osm adjusted with sucrose; [58]). For TEM, pediveliger larvae were postfixed for 1 h in 1 % OsO_4_ in buffer solution at 4 °C. For LM and TEM, larvae and juveniles were decalcified for 12 h at room temperature in 3 % ascorbic acid. Following dehydration in a graded ethanol series, specimens were embedded in Epoxy resin for TEM and glycol-methacrylate resin (Leica Historesin Kit, Germany) for LM. Serial 2–3 μm sections were obtained on a Leica RM2255 microtome (Leica, Wetzlar, Germany) for LM, and they were stained with hematoxylin and eosin or toluidine blue and basic fuchsin. Digital images were captured using a Nikon DS-Ri1 camera on a Nikon eclipse 80i microscope (Nikon Instech Co. Ltd, Kawasaki, Japan). For TEM, ultrathin sections were generated using a Leica Ultracut UCT microtome (Leica, Illinois, USA), mounted on copper slot-grids, contrasted with uranyl acetate and lead citrate, and analyzed using a Zeiss EM 900 electron microscope (Carl Zeiss, Oberkochen, Germany).

### Immunocytochemistry, Confocal Laser Scanning Microscopy (CLSM), and 3D Reconstruction

Specimens were fixed in 4 % paraformaldehyde in 0.1 M phosphate buffer (PB) for 1 h, followed by four rinses in the buffer solution. Samples were stored in 0.1 M PB containing 0.1 % NaN_3_ at 4 °C. Decalcification of larval and postmetamorphic individuals was performed in 0.05 M EGTA for 1 h at room temperature, with a maximum of 10 individuals per 5 ml in an embryo dish. For F-actin staining, specimens were permeabilized with PBS containing 2 % Triton-X 100 (PBT) overnight and then incubated in a 1:40 dilution of Alexa Fluor 488 phalloidin (Molecular Probes) in PBT for 24 h at room temperature in the dark. Cell nuclei were stained with DAPI (4′, 6-diamidino-2-phenylindole) (Invitrogen). Then, specimens were rinsed three times in PB for about 20 min each and mounted in Fluoromount G (Southern-Biotech, Birmingham, Alabama, USA) on standard microscope slides. Analysis and image acquisition were performed on a Leica TCS SP5 II confocal laser scanning microscope equipped with the software Leica Application Suite Advanced Fluorescence (LAS AF), Version 2.6.0 (Leica Microsystems, Wetzlar, Germany). Confocal image stacks were recorded with 0.3 μm step size along the z-axis and digitally merged as maximum intensity projections. 3D reconstructions were created from selected confocal stacks using the imaging software Imaris, Version 4.1 (Bitplane, Zürich, Switzerland).

## Additional files

Additional file 1:
**3D reconstruction of the veliger musculature in** ***Nodipecten nodosus*** **.** Anterior adductor is shown in orange, ventral larval retractors in green, dorsal velum retractor in blue, medio-dorsal velum retractor in red, medio-ventral velum retractor in pink, and ventral velum retractor in yellow. (MOV 2117 kb) Additional file 2:
**3D reconstruction of the pediveliger musculature of** ***Nodipecten nodosus*** **.** Adductor muscles are represented in orange, mantle muscles in blue, foot retractors in light green, and larval retractors in dark green. (MOV 15802 kb) Additional file 3:
**3D reconstruction of the postmetamorphic musculature of** ***Nodipecten nodosus*** **.** Adductor muscles are represented in orange, mantle muscles in blue, and foot musculature in green. (MOV 5918 kb)

## Abbreviations

CLSM: Confocal laser scanning microscopy
HE: Hematoxylin and eosin
LM: Light microscopy
PB: Phosphate buffer solution
PBT: Phosphate buffer solution containing Triton-X 100
TB: Toluidine blue and basic fuchsin
TEM: Transmission electron microscopy
